# Poly(2-oxazoline) Matrices with Temperature-Dependent Solubility—Interactions with Water and Use for Cell Culture

**DOI:** 10.3390/ma13122702

**Published:** 2020-06-13

**Authors:** Natalia Oleszko-Torbus, Marcelina Bochenek, Alicja Utrata-Wesołek, Agnieszka Kowalczuk, Andrzej Marcinkowski, Andrzej Dworak, Agnieszka Fus-Kujawa, Aleksander L. Sieroń, Wojciech Wałach

**Affiliations:** 1 Centre of Polymer and Carbon Materials, Polish Academy of Sciences, ul. M. Curie-Skłodowskiej 34, 41-819 Zabrze, Poland; noleszko@cmpw-pan.edu.pl (N.O.-T.); mbochenek@cmpw-pan.edu.pl (M.B.); autrata@cmpw-pan.edu.pl (A.U.-W.); akowalczuk@cmpw-pan.edu.pl (A.K.); amarcinkowski@cmpw-pan.edu.pl (A.M.); adworak@cmpw-pan.edu.pl (A.D.); 2 Department of Molecular Biology and Genetics, School of Medical Sciences in Katowice, Medical University of Silesia in Katowice, Medyków 18, 40-752 Katowice, Poland; afus@sum.edu.pl (A.F.-K.); alsieron@sum.edu.pl (A.L.S.)

**Keywords:** poly(2-oxazoline), matrice, thermoresponsiveness, cell culture, matrices, hydration, FDM, electrospinning

## Abstract

In this work, we studied the stability of matrices with temperature-dependent solubility and their interactions with water at physiological temperature for their application in cell culture in vitro. Gradient copolymers of 2-isopropyl- with 2-n-propyl-2-oxazoline (P(iPrOx-nPrOx)) were used to prepare the matrices. The comonomer ratio during polymerization was chosen such that the cloud point temperature (T_CP_) of the copolymer was below 37 °C while the glass transition (T_g_) was above 37 °C. The role of the support for matrices in the context of their stability in aqueous solution was examined. Therefore, matrices in the form of both self-supported bulk polymer materials (fibrillar mats and molds) and polymer films supported on the silica slides were examined. All of the matrices remained undissolved when incubated in water at a temperature above T_CP_. For the self-supported mats and molds, we observed the loss of shape stability, but, in the case of films supported on silica slides, only slight changes in morphology were observed. For a more in-depth investigation of the origin of the shape deformation of self-supported matrices, we analyzed the wettability, thickness, and water uptake of films on silica support because the matrices remained undeformed under these conditions. It was found that, above the T_CP_ of P(iPrOx-nPrOx), the wettability of the films decreased, but at the same time the films absorbed water and swelled. We examined how this specific behavior of the supported films influenced the culture of fibroblasts. The temperature-dependent solubility of the matrices and the possibility of noninvasive cell separation were also examined.

## 1. Introduction

Polymeric matrices for cell culture are bulk materials with a defined porosity and structure that contribute to the formation of new tissues for a wide range of biomedical applications [1]. These materials are usually applied in the form of thin films, gels, particles, foams, sponges, fibrous mats, or three-dimensional molds [2]. Generally, depending on the assumed structure, these materials can be obtained by the casting of polymer solutions, followed by stabilization and drying (films and gels), polymer–solvent phase separation or microsphere sintering (particles), porogen leaching (foams and sponges), electrospinning (fibrous mats), or rapid prototyping techniques (three-dimensional molds) [3]. Such matrices are designed for implantation directly into the body or for cell culture carried out in vitro [4,5,6,7].

Polymeric materials for cell culture should meet several design criteria: their surface should permit cell adhesion and growth, their porosity must ensure the diffusion of nutrients and metabolism products, and they must be nontoxic. It is also important that the mechanical and physicochemical properties of the polymer ensure the stability of the matrix during cell adhesion and growth. Since the polymeric matrices should remain stable under conditions mimicking the human body environment, they must have low affinity to aqueous solutions (hydrophobicity) to prevent dissolution or must be stabilized by crosslinking. On the other hand, after tissue formation in vitro (or its regeneration in vivo), it is often desirable to separate the matrices gradually from cultured cells. Thus, at this stage, high affinity to aqueous media that allows for matrix dissolution or the ability to degrade is highly important.

Different properties such the swelling, wettability, and degradation rate of the polymeric materials for cell culture have been investigated under aqueous conditions imitating the human body environment. For example, to determine the suitable conditions for osteoblast culture, Kim et al. [8] studied the affinity to water and degradation rate of nonwoven fibrous mats. It was found that electrospun nanofibers consisting of 40 wt.% high molecular weight poly(L-lactic acid) (PLA), 20 wt.% PLA-b-PEG-b-PLA, 25 wt.% low molecular weight random poly(lactic-co-glycolic) acid (PLGA, LA/GA = 50/50), and 15 wt.% lactide exhibited stable mechanical properties, an ideal biodegradation profile, and good hydrophilicity under aqueous conditions. Saito et al. [9] investigated the hydrophilicity, swelling capacity in PBS, and degradation rate of implants based on a triblock copolymer containing L-lactic acid, p-dioxanone and ethylene glycol. An optimal balance between the copolymer components led to the application of the implants for bone regeneration. Mozafari et al. [10] analyzed the properties of scaffolds constructed from bioactive glass, chitosan, and PLGA nanoparticles in PBS. The porosity, mechanical strength, swelling behavior and degradation rate of the matrices were easily controlled, and, thus, the obtained materials can be considered for use as a scaffold for bone tissue regeneration. The water absorption and diffusion characteristics were studied for blends of poly(L-lactic acid) and poly(hydroxybutyrate- co-hydroxyvalerate) with the addition of nanosized hydroxyapatite and were found to be promising for the applications of these blends in bone tissue engineering [11,12,13].

To date, studies conducted over several decades have shown that the most promising materials for cell culture are based on PLA [14], PLGA [15], polycaprolactone (PCL) [16], silk [17], collagen [18], hyaluronic acid [19], or chitosan [20]. In addition to exhibiting biocompatibility and good mechanical properties, materials based on these polymers are usually hydrophobic (or eventually crosslinked) and thus remain undissolved and stable during cell culture. The degradation of these polymers generally proceeds at a good rate, allowing the separation of cells from the polymer after culture.

The application of polymers with thermoresponsive properties may provide an interesting alternative to the use of the abovementioned materials for cell culture in vitro. Such polymers would provide materials with temperature-dependent solubility (TDS-materials). Above a certain temperature, called the cloud point temperature (T_CP_), thermoresponsive polymer is dehydrated and remains undissolved in aqueous medium due to the strong interactions between the polymer chains [21]. Taking into account the cell culture conditions, the T_CP_ of a thermoresponsive polymer must be tailored to below 37 °C. At this temperature, the TDS-material based on this polymer will remain dehydrated and undissolved. Additionally, the glass transition (T_g_) of the polymer should be tailored to be above 37 °C, so that the polymer will remain in the glassy state, ensuring the stability of the matrices during cell culture. After cell proliferation, the cell culture temperature would be decreased to below T_CP_. Under these conditions, the interactions between the thermoresponsive polymer chains and water molecules dominate, and the polymer undergoes dissolution [22]. The polymer could then be simply washed out from the culture environment so that no synthetic product will remain within cultured cells. To date, TDS-materials have not been applied as cell culture matrices, and, therefore, their stability under conditions imitating the human body environment (when the polymer is dehydrated) and their dissolution rate after the temperature decrease are unknown.

Many thermoresponsive polymers have been described in the literature, such as poly(N-substituted acrylamides)s (mostly PNIPAM) [23]; poly[oligo(ethylene glycol) methacrylate]s [24]; polymers with pendant hydroxyl groups, including poly(vinyl alcohol) or polyglycidol derivatives [25,26]; and polyethers, including PEO and PPO [27]. Recently, there has been resurgent interest in poly(2-oxazoline)s (POx), thermoresponsive polymers, which are known as thermoresponsive pseudopeptides, due to their potential application in medicine and biotechnology [28,29,30,31,32,33,34,35,36]. POx are nontoxic, biocompatible polymers [37,38] that do not accumulate in tissue but are rapidly cleared from the bloodstream [39]. The polymerization process, the composition of 2-oxazoline (co)polymers, and thus their properties can be easily controlled. The T_CP_ of POx can be modulated by the balance between the hydrophilic and hydrophobic elements in the chain [40,41,42,43]. Furthermore, the T_g_ of POx can be simply adjusted by the appropriate balance between elements with different stiffnesses and mobilities in the chains [44]. In our previous studies, we showed that the use of thermoresponsive POx nanolayers covalently attached to a solid support facilitated the culture of skin cells and did not interfere with cell morphology, phenotype or gene expression [45,46]. Encouraged by these results, we developed self-supported matrices in the form of nonwoven fibrous mats and multilayer molds based on poly(2-isopropyl-2-oxazoline) (PiPrOx) and gradient copolymers of 2-isopropyl- with 2-n-propyl-2-oxazoline (P(iPrOx-nPrOx)) [47]. Both the T_CP_ and T_g_ of the polymers were adjusted with the goal of being able to apply them in the construction of matrices with temperature-dependent solubility for cell culture in vitro. Copolymers of 2-isopropyl- with 2-n-propyl-2-oxazoline were found to be more easily processed and more stable upon processing than PiPrOx. Moreover, matrices based on these copolymers exhibited good physicochemical properties (elasticity and low crystallinity), which are important for ensuring the stability of the material during cell culture.

This work is a continuation of our previous research and investigates the behavior of P(iPrOx-nPrOx) matrices in an aqueous environment [47].

Here, we investigated the properties of P(iPrOx-nPrOx) matrices under aqueous conditions imitating the human body environment, as well as their temperature-dependent solubility and their applicability as TDS-materials for cell culture in vitro. Self-supported nonwoven fibrous mats, multilayer molds, and thin films supported on silica slides were prepared. The stability of the prepared matrices in aqueous solutions at 37 °C was analyzed. Additionally, the morphology, wettability, thickness, and water uptake of P(iPrOx-nPrOx) films were studied under these conditions. The dissolution rate of the matrices with the lowering of temperature was evaluated. Dermal fibroblasts were cultured on P(iPrOx-nPrOx) films, and their adhesion, morphology, and proliferation rate were assessed. Temperature-dependent cell detachment from the matrices based on the transition temperature was investigated.

## 2. Materials and Methods

Materials: Isobutyronitrile (99.6%, Sigma-Aldrich, Steinheim, Germany), n-butyronitrile (>99%, Fluka, Steinheim, Germany), 2-aminoethanol (99%, Aldrich, Steinheim, Germany), cadmium acetate (>98%, Fluka, Steinheim, Germany), methyl 4-nitrobenzenesulfonate (99%, Aldrich, Steinheim, Germany), and propylamine (>99%, Aldrich, Steinheim, Germany) were used as received. Acetonitrile (ACN, 99.9%, POCH, Gliwice, Poland) was dried over CaH_2_ and distilled under dry argon. Polished prime silica slides (Cemat Silicon S.A, Warszawa, Poland) with a thickness of ~500 µm and a SiO_2_ layer of 3 nm were cut into 1 cm^2^ pieces. Dulbecco’s Modified Eagle’s Medium (DMEM), phosphate-buffered saline (PBS), and laminin were purchased from Sigma Aldrich. Penicillin/streptomycin/amphotericin B mix, fetal bovine serum (FBS), L-glutamine and trypsin/EDTA (10×) were purchased from PAN Biotech. Alamar blue assay reagent (DAL1100) was purchased from Invitrogen.

Synthesis of monomers: The protocol for the synthesis of monomers was the same as that described previously [48]. Briefly, isobutyronitrile or n-butyronitrile was mixed in an equimolar amount with 2-aminoethanol and heated under reflux in the presence of cadmium acetate. The raw monomers were dried over KOH, distilled under reduced pressure, dried over CaH_2_, and then distilled again.

Polymerization: The procedure for polymerization was the same as that described in [49]. Briefly, methyl 4-nitrobenzenesulfonate was dissolved in dried acetonitrile, and the freshly distilled monomers were added. The theoretical degree of polymerization was assumed to be 450. Polymerization was carried out at 75 °C. The living polymer chains were terminated by the addition of n-propylamine. After evaporation of acetonitrile, the polymer was dissolved in deionized water and then dried by lyophilization. The polymer was named P(iPrOx-nPrOx).

Characterization of the polymer: The molar mass and dispersity (*Ð*) of P(iPrOx-nPrOx) were determined using a gel permeation chromatography system with a multiangle laser light scattering detector (GPC MALLS, Santa Barbara, CA, USA) (DAWN EOS, Wyatt Technologies, λ= 658 nm) and a refractive index detector (WGE DR Bures, Dallgow, Germany) (n-1000 RI WGE DR Bures, λ= 620 nm). Measurements were carried out in DMF (with 5 mmol/L of LiBr; flow rate of 1 mL/min) using 10 µm PSS 100, 1000, and 3000 Å GRAM columns. The refractive index increment (*dn/dc*) was independently measured in DMF and was equal to 0.071. The composition of P(iPrOx-nPrOx) was calculated from the ^1^H NMR spectra. The spectrum was recorded using a Bruker Ultrashield spectrometer operating at 600 MHz in CDCl_3_ as the solvent. The cloud point temperature was determined based on turbidity measurements (λ=500 nm) of the aqueous solution of P(iPrOx-nPrOx) (5 g/L), monitored as a function of temperature using a Specord 200plus UV-Vis spectrophotometer (Analytik Jena, Jena, Germany). T_CP_ was defined as the temperature at which the transmittance reached 50% of its initial value. The glass transition of P(iPrOx-nPrOx) was determined based on the DSC measurement performed using a TA-DSC Q2000 apparatus (TA Instruments, Newcastle, DE, USA). The temperature range from −50 to 250 °C and a heating rate of 10 °C/min were applied.

Preparation of matrices: P(iPrOx-nPrOx) matrices were obtained in the form of nonwoven fibrous mats, multilayer molds of cylindrical and cubical shape, and thin films supported on silica slides. Nonwoven fibrous mats were obtained using the basic set-up for electrospinning, which was composed of a syringe pump (KD Scientific, Holliston, MA, USA), a high voltage power supply (Gamma High Voltage Research, Ormond Beach, FL, USA), and a flat steel plate collector covered with aluminum foil. To obtain three-dimensional molds, a Bioscaffolder system (SYSENG, Salzgitter-Bad, Germany) was used. The fibrous mats used in this study were obtained by applying a concentration of P(iPrOx-nPrOx) in water of 45 wt.% and a flow rate of 1 mL/h. Multilayer molds were obtained at 160 °C by applying a pressure of 5 bar during extrusion through the nozzle and a screw rotation of 160 rpm. The optimization of the conditions during electrospinning and the fused deposition modeling of P(iPrOx-nPrOx), both with schematic representations of the systems, are described in detail in [47]. P(iPrOx-nPrOx) thin films were obtained by drop-casting of the aqueous polymer solutions onto silica slides and drying at 40 °C for 4 days. Film 1 was obtained by casting of the 4 wt.% P(iPrOx-nPrOx) solution (1.5 mL), Film 2 was obtained by casting of the 6.5 wt.% solution (1.5 mL), and Film 3 was obtained by casting of the 6.5 wt.% solution (0.2 mL). The scheme presenting the preparation of the matrices is shown in Figure S1.

Characterization of matrices: First, the matrices were analyzed in a dry state. Then, the matrices were incubated in water at 40 °C for 2 h and gently blown with a stream of argon. For simplicity, the matrices treated in this manner were named “samples after incubation”.

The morphology of the matrices was visualized by atomic force microscopy (AFM) and scanning electron microscopy (SEM). Microscopic observations were carried out for P(iPrOx-nPrOx) films supported on silica slides and fibrillar mats. A multimode AFM microscope equipped with a NanoScope 3D controller (Veeco Instruments Inc., San Jose, CA, USA) and piezoelectric scanner was used. Phosphorus-doped silicon cantilevers (Model TESP, BRUKER, Billerica, MA, USA) with a length of 125 μm and a tip height of 15–20 μm were applied. Micrographs were recorded in the tapping mode. The reported RMS roughness values were measured over the 5 µm × 5 µm surface and are the average of two measurements. SEM analysis was performed using a Quanta 250 FEG (FEI Company, Hillsboro, OR, USA). The matrices were observed under low vacuum (80 Pa) with an acceleration voltage 5 kV from secondary electrons collected by a Large Field Detector (LFD).

The wettability of the P(iPrOx-nPrOx) films supported on silica slides was measured using a CAM101 goniometer equipped with a temperature control unit (Intelligent digital controller OMRON 5EGN) connected to a thermostatically controlled chamber. The contact angle measurements were performed following the sessile drop method. A water droplet was placed on the surface and was registered for 150 s. The measurements were performed at room temperature for films in a dry state and at 40 °C for films after incubation. The contact angle values were calculated as average from three measurements.

The thickness of the P(iPrOx-nPrOx) films on silica slides was analyzed by SEM. The matrices were placed in the vertical position in a SEM sample holder for cross-section analysis, and the thickness was evaluated using the morphology differences between the polymer film and silica slices. The thickness values were averaged from three measurements.

The water uptake by P(iPrOx-nPrOx) films was monitored by the increase in the film weight. The films were first weighed precisely and then immersed in water at 40 °C for 2 h. Then, the films were removed, gently blown under a stream of argon and weighed. Then, the films were weighed again after 1 and 24 h of drying at 40 °C. The weight gain was calculated from three measurements for each sample as weight of absorbed water/weight of the dry polymer layer. The presence of absorbed water was monitored by FT-IR for the films directly after incubation and after 1 and 24 h of the subsequent samples drying at 40 °C. The spectra were recorded using an FTIR 6700 spectrometer (Nicolet, Thermo Scientific) equipped with a diamond crystal Smart OrbitTM accessory working in the attenuated total reflection mode (ATR). All of the spectra were acquired between 4000 and 500 cm^−1^ with 128 accumulations and were evaluated using the OMNIC™ software. The spectrum of a freshly cleaned silica plate was recorded prior to the measurements and was used as the background reference.

Cell culture: DMEM and PBS were dissolved in sterile distilled water in accordance with the manufacturer’s instructions. Penicillin/streptomycin/amphotericin B mix, FBS, L-glutamine, and trypsin/EDTA (10×) were added to the culture medium to achieve their proper final concentrations prior to use. L-glutamine was added to the culture medium every 2 weeks. Cells were seeded at a density of 5 × 10^4^ cells per single well of the 24-well culture plate in a cell culture medium consisting of DMEM supplemented with 10% FBS, 1% L-glutamine, penicillin, streptomycin, and amphotericin B on P(iPrOx-nPrOx) films and TCPS as the control at 37 °C and 5% CO_2_. Adhesion and proliferation assays were performed with the Alamar blue assay reagent used as a 10% solution. These assays were performed after 2.5, 4, 8, 24, 48, and 72 h of cell culture and after an overnight incubation at 37 °C for P(iPrOx-nPrOx) films with the addition of laminin (2.42 µg/mL), as previously described [50]. For this purpose, after each time point, medium was removed and fresh, prewarmed DMEM containing 10% Alamar blue (200 μL) was added to each well and incubated for 1 h at 37 °C. After incubation, the medium (100 μL) was collected and measured spectrophotometrically. The number of cells in the culture well was estimated from a calibration curve based on Alamar blue fluorescence and was statistically analyzed for P(iPrOx-nPrOx) films and TCPS. Cell morphology was visualized after each time point using an inverted light microscope (OLYMPUS, Tokyo, Japan) equipped with a digital camera (XC50, Olympus, Tokyo, Japan). The temperature-dependent solubility of the matrices and the possibility of noninvasive cell separation was tested after 72 h of cell culture. For this purpose, the culture temperature was decreased to 18 °C and the films were incubated under this condition for 60 min.

## 3. Results

### 3.1. Behavior of TDS-Matrices in Water

For the formation of the matrices, the gradient copolymer of 2-isopropyl- with 2-n-propyl-2-oxazoline (P(iPrOx-nPrOx)) was used. Since the matrices were designed as TDS-materials for cell culture, the monomer ratio during polymerization was chosen such that the T_CP_ of P(iPrOx-nPrOx) was below 37 °C, while the T_g_ was above this temperature (Table 1). We assumed that, under these conditions, the polymer would remain dehydrated and glassy (not plastic), ensuring the stability of the matrices during cell culture.

The P(iPrOx-nPrOx) matrices in the form of self-supported nonwoven fibrous mats, self-supported multilayer molds, and films supported on silica slides were obtained, and their properties were analyzed under aqueous conditions to apply them for cell culture in vitro. The optimization conditions for the preparation of self-supported matrices and their physicochemical properties in bulk have been presented elsewhere [47]. For the preparation of the films supported on silica slides, drop-casting of the P(iPrOx-nPrOx) solution was used, and three batch of samples were obtained, named Film 1 (1.5 mL of 4 wt.%), Film 2 (1.5 mL of 6.5 wt.%), and Film 3 (0.2 mL of 6.5 wt.%).

**Figure 1:**
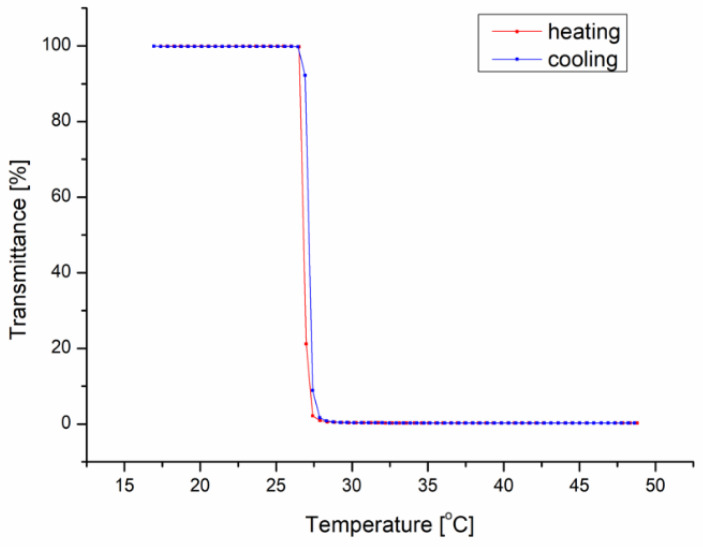
Sample temperature-transmittance curves for the solution obtained after the dissolution of the P(iPrOx-nPrOx) mold.

All of the obtained matrices underwent dissolution within several minutes when placed in water at 20 °C (below T_CP_). The solution after the dissolution of the matrices exhibited a T_CP_ of 27 °C (Figure 1).

**Figure 2:**
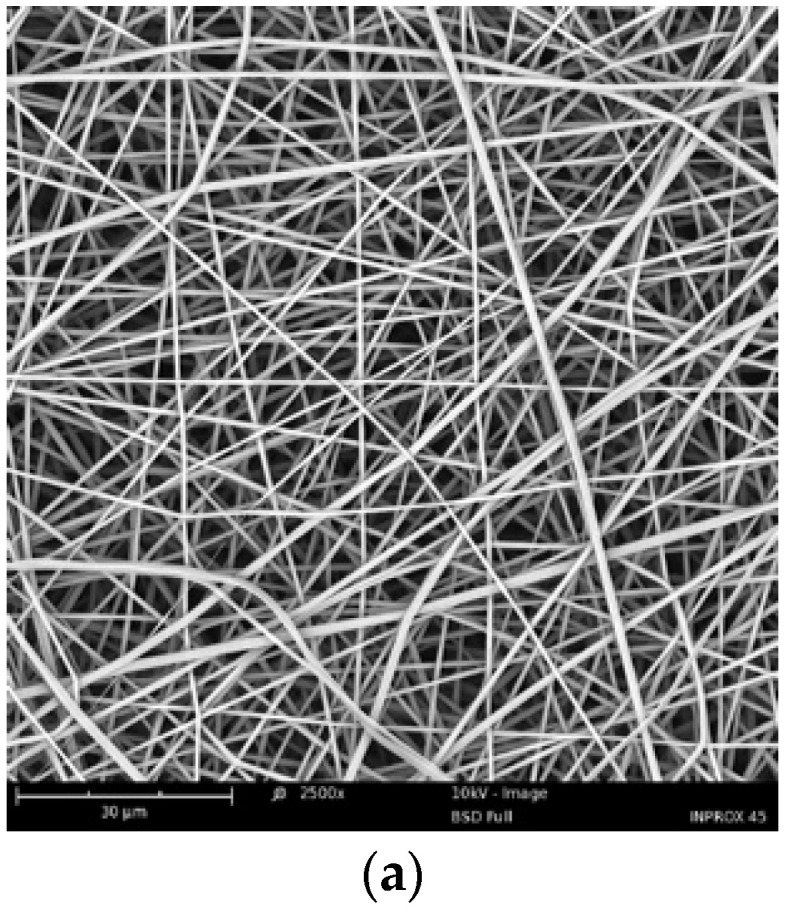

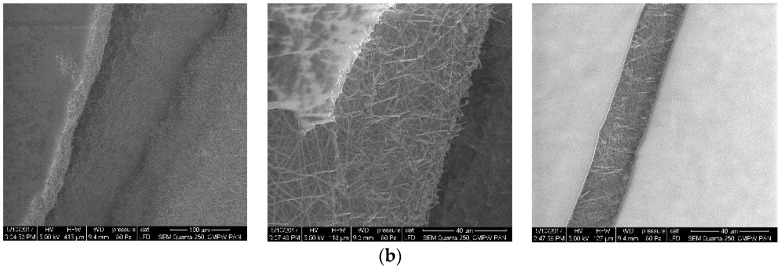
SEM micrographs of the P(iPrOx-nPrOx) mat: (**a**) in the dry state; and (**b**) after incubation in water at 40 °C for 2 h.

Then, all of the matrices were placed in water at 40 °C (above T_CP_) and were incubated under this condition for 2 h. After this time, the matrices remained undissolved, but, in the case of the self-supported fibrous mats and three-dimensional molds, the undesired loss of shape stability was observed. The fibrous mats were rolled up (see the SEM micrographs of P(iPrOx-nPrOx) mats during rolling in Figure 2). Additionally, the regular shape of the molds was distorted and they collapsed into shapeless clods (see Figure S2).

**Figure 3:**
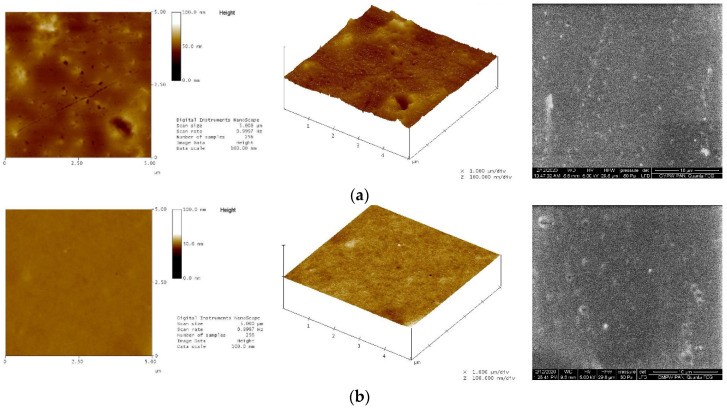
AFM and SEM micrographs of Film 2: (**a**) in the dry state; and (**b**) after incubation in water at 40 °C.

For P(iPrOx-nPrOx) films supported on silica slides, their shape stability was preserved, but some slight changes in morphology were observed by AFM and SEM. The films in a dry state exhibited a slightly irregular surface with root mean square roughness (RMS) values of approximately 1.26, 3.78, and 1.01 nm for Films 1–3, respectively, as obtained by AFM. After incubation in water at 40 °C for 2 h, the films became smooth, and RMS values of approximately 0.35, 0.36, and 0.77 nm were obtained for Films 1–3, respectively. Sample micrographs of Film 2 in the dry state and after incubation in water are presented in Figure 3 (micrographs of Films 1 and 3 are shown in Figure S3).

We expected that at an elevated temperature, i.e., above the T_CP_ but below the T_g_ of the P(iPrOx-nPrOx), polymer chains should be poorly solvated and should repel water from the matrix, ensuring the stability of the material. However, the loss of shape in the case of self-supported matrices was observed, possibly because of the softening of the material due to the probable interactions of the polymer chains with water, which are unexpected under these conditions. To clearly explain the origin of the shape deformation and to determine the factors affecting this process, we sought to analyze the interactions between the thermoresponsive P(iPrOx-nPrOx)-based layers and water above T_CP_. For this analysis, P(iPrOx-nPrOx) films drop-casted on silica slides were chosen because, in this case, due to the presence of a solid support, the stability of the films after incubation in water above T_CP_ was preserved, even though the outermost layer was slightly smoothed. The wettability, thickness, and water uptake were analyzed for the films in the dry state at room temperature and after incubation in water at 40 °C. Finally, we analyzed how the nature of the thermoresponsive P(iPrOx-nPrOx)-based layers influences cell culture. Most importantly, we examined whether it was possible to separate cells from the matrix after culture by merely dissolving the polymer.

#### 3.1.1. Wettability of P(iPrOx-nPrOx) Films

**Figure 4:**
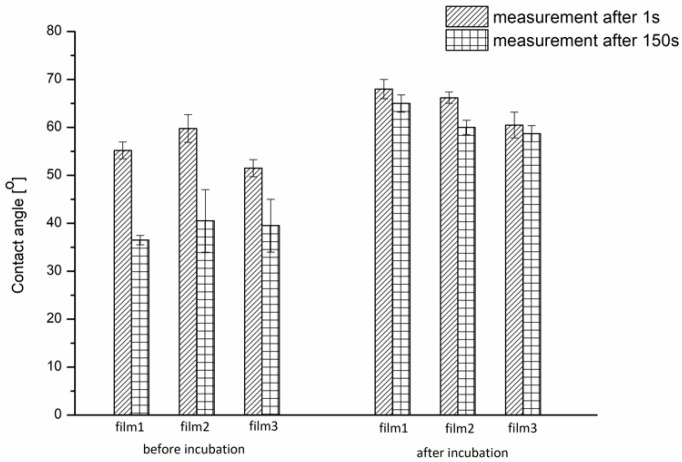
**Θ** values for P(iPrOx-nPrOx) films in the dry state at room temperature and after incubation in water at 40 °C and measured at 40 °C.

The affinity to water of the P(iPrOx-nPrOx) films was studied by contact angle measurements (Θ) carried out using the sessile drop method. The wettability of the P(iPrOx-nPrOx) films in the dry state was analyzed at room temperature, while in the case of films after incubation in water at 40 °C, the affinity to water was measured at 40 °C. The values are presented in Figure 4.

For dry P(iPrOx-nPrOx) films at room temperature (below T_CP_), good wettability was observed due to the high affinity of the copolymer to water under these conditions. Θ values between 50° and 60° were observed. During the 150 s of the measurement, the water droplet soaked into the dry polymer films. The difference between the value measured after the Second 1 and 150 of the contact of the water droplet with the polymer layer was approximately 20° for Film 1 and Film 2 and ~10° for Film 3. For the films after incubation in water above T_CP_, the contact angle values measured at 40 °C were as high as 70°, indicating decreased wettability due to the domination of the polymer chains interactions over the polymer–water interactions. During the 150 s of the measurement at 40 °C, the permeation of the water droplet into the polymer film was not as significant as that for dry films measured at room temperature, confirming the predominant role of the polymer chain interactions above T_CP_. On the other hand, microscopic analysis (SEM and AFM) revealed that, despite these interactions, a slight smoothing of the outer surface of the P(iPrOx-nPrOx) films after incubation occurred, indicating some contribution of water. It appears that the polymer is likely gradually plasticized over time by water to form a gel-like layer. For the thinnest Film 3, the contact angle value measured above T_cp_ after incubation in 40 °C is the lowest, which is probably related to the highest hydration of the whole film Further studies of the thickness and water uptake of this films supported this observation.

#### 3.1.2. Thickness of P(iPrOx-nPrOx) Films

P(iPrOx-nPrOx) films in the dry state and after incubation in water at 40 °C for 2 h were observed by scanning electron microscopy. The thickness measurements were carried out using an SEM sample holder for cross-section analysis. The matrices were placed in a vertical position, and their thickness was measured using the morphology differences between the polymer film and silica slices. The obtained values are presented in Table 2. The SEM micrographs are included in the Supplementary Materials (Figure S4).

The thickness of P(iPrOx-nPrOx) films in the dry state was varied by applying polymer solutions with different amounts and concentrations during drop-casting. Using a more concentrated P(iPrOx-nPrOx) solution, a greater film thickness was obtained (Film 2, 6.5 wt.%) than for the film prepared by the casting of the less concentrated copolymer solution (Film 1, 4 wt.%). Additionally, by applying greater amounts of the polymer solution, a thicker layer was obtained (Film 2, 1.5 mL) than for the sample prepared by applying a smaller amount of solution at the same concentration (Film 3, 0.2 mL).

After the 2-h incubation of P(iPrOx-nPrOx) films in water above T_CP_, the film thickness increased to 70 ± 15 µm in case of Film 1, to 220 ± 20 µm in the case of Film 2, and to 30 ± 5 µm in the case of Film 3. These results indicate that water was absorbed by the films, leading to the increase in the thickness. However, these results were rather unexpected because the simultaneous wettability studies revealed the domination of the polymer chain interactions over the polymer–water interactions above T_CP_. The lowest increase in thickness relative to the initial thickness was observed in the case of the thickest layer in the dry state (Film 2). To confirm the presence and the amounts of water absorbed within the P(iPrOx-nPrOx) films, water uptake studies were performed.

#### 3.1.3. Water Uptake by P(iPrOx-nPrOx) Films

**Figure 5:**
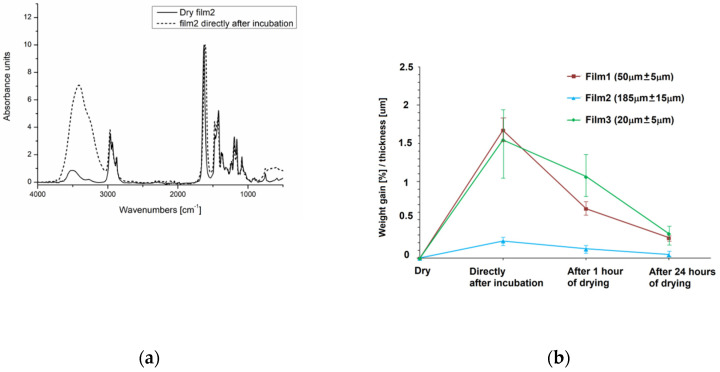
(**a**) FT-IR spectra of Film 2 in the dry state and directly after incubation in water at 40 °C; and (**b**) the weight gain of the P(iPrOx-nPrOx) films relative to their thickness after incubation.

For the water uptake studies, composition analysis of P(iPrOx-nPrOx) films was performed by FT-IR, and the weight gain of the layers after incubation in water at 40 °C was analyzed. Composition analysis was monitored by FT-IR for the films in the dry state, directly after incubation in water at 40 °C and after 1 h and after 24 h of subsequent sample drying at 40 °C. The sample FT-IR spectra of Film 2 in the dry state and directly after incubation are shown in Figure 5a.

In the spectrum of the dry film, characteristic absorption bands of polyoxazoline are observed. These bands are located at 2963–2870 cm^−1^ (CH_2_ stretch), 1625 cm^−1^ (C=O stretch), 1420–1470 cm^−1^ (C–H deformation), 1362–1380 cm^−1^ (C-N stretch), 1160–1200 cm^−1^ (C–H deformation), and 1088 cm^−1^ (C–O stretch). The absorption band at 3520 cm^−1^ that is observed in the spectra of the film in the dry state is due to the stretching vibration of O–H from the residual water in the polymer. This conclusion is supported by the fact that this band is absent in the spectrum of P(iPrOx-nPrOx) dried in high vacuum (see the spectrum in Figure S5), which means that drying the films during preparation (four days at 40 °C) does not completely remove water from their interior and explains the significant affinity of P(iPrOx-nPrOx) to water. The band of the stretching vibrations of O–H observed in the FTIR spectra of the film after incubation was shifted to 3300 cm^−1^, and its intensity was significantly higher than that for the dry films. Additionally, the absorption band ascribed to C=O stretching was also shifted from 1625 to 1605 cm^−1^. All of these results confirm the formation of hydrogen bonds and thus the presence of a significant amount of water within the polymeric layers. The films after 1 h and 24 h of drying still contain a small but noticeable amount of water (spectra not shown).

For the weight gain analysis, films in the dry state were precisely weighed and then were weighed directly after incubation in water at 40 °C and after 1 and 24 h of subsequent sample drying at 40 °C. Based on the weight gain, the amount of absorbed water was determined. Figure 5b presents the weight gain of the films with respect to their thickness directly after incubation and after 1 and 24 h of drying. For all of the P(iPrOx-nPrOx) films after incubation, a significant weight gain was observed. Considering the thickness of the samples, it is observed that thinner layers (Films 1 and 3) absorbed significantly more water than the thickest Film 2. Drying of swollen films at 40 °C in air led to a clear decrease in weight associated with water evaporation until the almost complete drying of the films after 24 h.

To summarize, P(iPrOx-nPrOx) films incubated in water above T_CP_ exhibited decreased wettability at 40 °C, indicating the domination of polymer chain interactions over polymer–water interactions. Nevertheless, polymer–water interactions could not be completely excluded, even though the temperature is above T_CP_, which was demonstrated by water absorption into the films (confirmed by an increase in thickness and composition analysis) and the slight smoothening of the outer surface of the films, leading to a gel-like layer (microscopic observations).

Okano et al. reported interesting observations on the interactions of thermoresponsive polymer chains on the surface with water, depending on layer thickness [51]. They considered PNIPAM crosslinked layers of different thicknesses that were covalently attached to glass coverslips or tissue culture polystyrene (TCPS) plates and that were prepared by electron-beam irradiation. It was found that, for several nanometer thick layers, above T_CP_, the interactions between the polymer chains dominated over the polymer–water interactions within the entire layer, so that dehydration and repelling of water could be observed. However, for thicker layers with thicknesses of at least of dozens nanometers, at these conditions only the part of PNIPAM chains that was at the interface with a solid substrate was poorly solvated, while the part of the layer within the outermost region interacted strongly with water. The PNIPAM chains at the interface with the solid substrate were defined as highly dehydrated and aggregated, the chains toward the outermost surface were defined as chains of restricted hydration and mobility, and the most external part of the chains were hydrated and mobile.

It is likely that a similar model can be applied to the behavior of the thermoresponsive P(iPrOx-nPrOx) films drop-casted on silica slides. Even though the films are immersed in water preheated above T_CP_, water absorption occurs to a certain degree. Taking diffusion into account, a gradient of polymer layer hydration occurs: the part of the layer at the interface with solid support is poorly solvated and the part of the layer in the outermost region is hydrated. Considering films with different thicknesses, the lowest amount of water was absorbed in the case of the thickest film (Film 2), most likely due to the dehydration and aggregation of the P(iPrOx-nPrOx) chains at the interface with the solid support. The hydration of the part of the P(iPrOx-nPrOx) layers despite of the elevated temperature most likely led to the shape deformation of the matrices that were prepared without the support (self-supported mats and molds). Thus, it appears that the presence of a solid support for matrices with temperature-dependent solubility is crucial for their stability. Alternatively, the addition of some stabilizing substance to the self-supported matrices, such as ceramics (e.g., hydroxyapatite or bioglass), carbon materials, or composites, can be considered because such modifications can reduce or even prevent the effect of deformation and strengthen the structure of the material. For example, such an effect was observed for the chitosan/58S-bioactive glass scaffolds containing PLGA nanoparticles [10]. It was shown that the mechanical strength of the structure was increased by increasing the weight percentage of bioactive glass. It was also found that the swelling behavior of the scaffolds was reduced and that the porosity decreased with the increasing content of this compound. Although such strengthening of the structure is also likely for P(iPrOx-nPrOx) matrices, this issue requires further study. It should be noted that such modifications of self-supported matrices intended for application as TDS-materials can lead to undesirable changes in their solubility properties.

Since we sought to analyze how the nature of thermoresponsive P(iPrOx-nPrOx)-based matrices influences cell culture in vitro, we used films drop-casted on silica slides because, due to the presence of solid support, their stability after incubation in water above T_CP_ was preserved.

### 3.2. Cell Culture on P(iPrOx-nPrOx) Films

**Figure 6:**
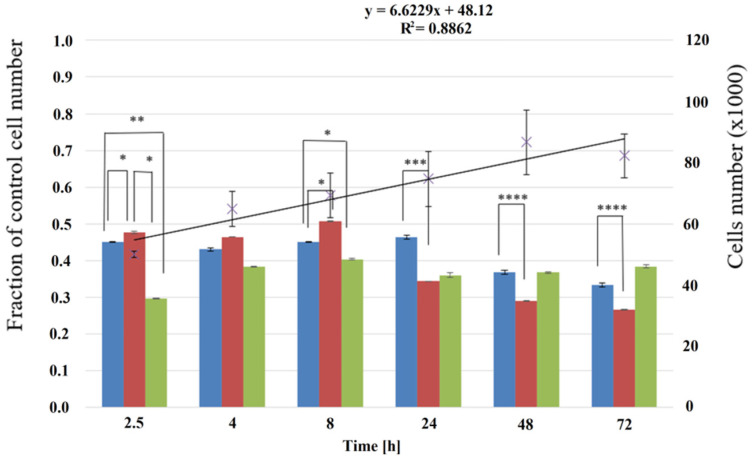
Percentage of fibroblasts cultured on P(iPrOx-nPrOx) films and TCPS. The fraction of control cells and cell numbers were assayed from 2.5 to 8 h for adhesion and spreading and for proliferation; the assays were conducted after 24, 48, and 72 h. The results for Films 1–3 are shown in blue, red, and green, respectively. The line presents the changes in the number of cells cultured on TCPS from 2.5 to 72 h of the incubation. Statistical analysis was performed using two-tailed Student’s test. Stars indicate *p*-values: * *p* < 0.05, ** *p* < 0.01, *** *p* < 0.001, **** *p* < 0.0001. Values are mean  ±  SD (n = 3).

P(iPrOx-nPrOx) films were used as matrices for the culture of fibroblasts. To prevent films from dissolution (T_CP_ of P(iPrOx-nPrOx) = 27 °C), all procedures connected with the preparation and analysis of cell culture were carried out above this temperature. Cells were suspended in a culture medium prewarmed to 37 °C and seeded at a density of 50,000 cells per each well of a 24-well culture plate. To observe the cells, calculate their number, and determine their morphology after a set time of culture, medium with non-adsorbed cells was removed at 37 °C (not at room temperature, as it is usually performed). The number of fibroblasts after 2.5, 4, 8, 24, 48, and 72 h compared to the control (tissue culture polystyrene-TCPS) was calculated based on a spectrophotometrical analysis carried out with the Alamar blue test (Figure 6). The morphology of the cells after these time periods was also analyzed under an inverted light microscope (OLYMPUS, Tokyo, Japan).

For all films, the cell number decreased compared to the number of cells cultured on TCPS (control). The greatest number of cultured fibroblasts was observed on Film 2 after 8 h of incubation, but then the number of cells dropped until 72 h by almost half. Films 1 and 2 were slightly better for cultured cells at the beginning of culture than Film 3. For the first 24 h for Film 1 and after 8 h for Film 2, the fraction of cultured cells did not change compared to the cell numbers cultured on TCPS, but, in further cell culture, it decreased until almost 1/3 of the cell number cultured on TCPS was reached.

**Figure 7:**
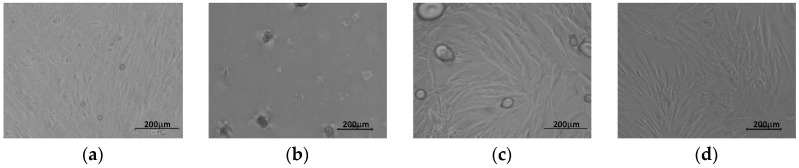
Morphology of fibroblasts cultured on P(iPrOx-nPrOx) after 72 h of cell culture: (**a**) Film 1; (**b**) Film 2; (**c**) Film 3; and (**d**) TCPS.

The morphology of fibroblasts cultured on P(iPrOx-nPrOx) films differed from that of cells proliferated on TCPS (Figure 7).

The cells were rather round-shaped and gathered into clusters. After 72 h, a spindle-like morphology of fibroblasts was observed for Films 1 and 3. For Film 2 (the thickest film), clusters of round-shaped cells could be seen. Laminin, which is a peptide of the extracellular matrix synthesized by epithelial cells in human skin, was added to the culture medium to improve the adhesion of fibroblasts that proliferated on Film 2; however, no significant improvement in the number of cells or morphology was observed (Figure S6).

The adhesion and proliferation of fibroblasts on P(iPrOx-nPrOx) films was not efficient compared to the control, likely due to the specific nature of thermoresponsive polymer layer. Although the films were non-solvated at the interface with the silica substrate, they remained hydrated at the outermost region and still contained absorbed water, leading to a gel-like layer. Such a structure of the layer does not appear to be entirely beneficial for the proper adhesion and formation of cell-to-cell junctions compared to the dehydrated in full volume polymer nanolayers described in the literature.

**Figure 8:**
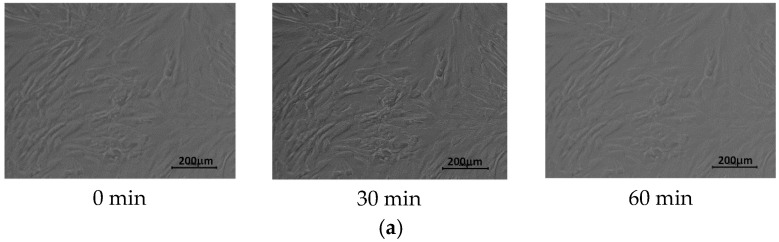

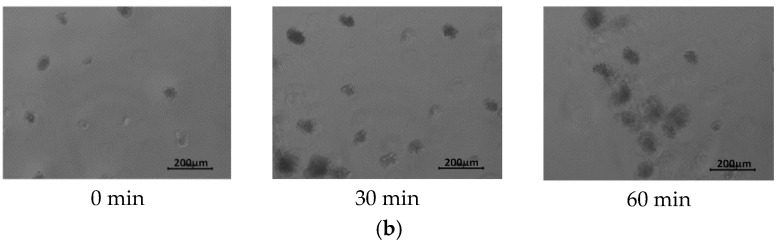
Morphology of fibroblasts at 18 °C on: (**a**) TCPS; and (**b**) Film 2.

The temperature-dependent solubility of the matrices and possibility of noninvasive cell separation was also tested. After 72 h of cell culture, the culture temperature was decreased to 18 °C, which is below the T_CP_ of P(iPrOx-nPrOx). Microscopic observations revealed that, during 60 min of incubation at 18 °C, fibroblasts moved significantly compared with cells prior to the temperature decrease. This result confirmed that the polymer matrices were dissolving during this time and that the cells lost their anchor surface. The cell clusters moved within the suspension of the polymer solution. When the temperature of the medium containing the polymer and cells was increased again to 37 °C, the solution became turbid, confirming the additional dissolution of the P(iPrOx-nPrOx) matrices. By contrast, a lack of cell mobility at 18 °C was observed in the case of fibroblasts cultured on TCPS. Figure 8 presents micrographs of fibroblasts on TCPS and Film 2 at 18 °C.

## 4. Conclusions

In this work, the preparation of matrices with temperature-dependent solubility (TDS-materials) with potential for application in cell culture in vitro was carried out. The gradient copolymer of 2-isopropyl- with 2-n-propyl-2-oxazoline was used to prepare matrices both in the form of self-supported bulk polymer materials (fibrillar mats and molds) and as polymer films supported on silica slides. The crucial issue investigated in the work was the behavior of the obtained matrices under aqueous conditions imitating the human body environment. Although the dry P(iPrOx-nPrOx) matrices submerged in water at a temperature above T_CP_ remained undissolved, the loss of shape stability was observed for self-supported mats and molds. For films supported on silica slides, only slight changes in morphology were visible. These films, after incubation in water above T_CP_, exhibited decreased wettability at 40 °C, indicating the domination of polymer chains interactions over polymer–water interactions. On the other hand, the unexpected increase in the thickness of the films after incubation in water above T_CP_ was observed, indicating the absorption of water. The formation of hydrogen bonds and thus the presence of a significant amount of water within P(iPrOx-nPrOx) films was confirmed by the analysis of the layer composition. Films 1 and 3 absorbed significantly more water relative to the film thickness than the thickest Film 2, which indicated a probable gradient of hydration within the polymer layer: the part of the layer at the interface with the solid support was poorly solvated while the part of the layer in the outermost region was hydrated. Studies of the morphology, wettability, thickness, and water uptake of P(iPrOx-nPrOx) films supported on silica slides confirmed that the polymer–water interactions could not be completely neglected even for temperature above T_CP_. The hydration of the part of the P(iPrOx-nPrOx) layers despite the elevated temperature was the most likely origin of the shape deformation for the matrices prepared without support (self-supported mats and molds). Thus, it appears that the presence of a solid support for matrices with temperature-dependent solubility is crucial for their stability. Finally, we analyzed how the nature of the P(iPrOx-nPrOx) films influences the culture of fibroblasts. The performed experiments revealed that fibroblast adhesion and the fibroblast proliferation rate on partly hydrated P(iPrOx-nPrOx) films decreased compared to those on TCPS. Even the addition of laminin as a component of the basal lamina that influences migration, differentiation, and adhesion of cells did not enable obtaining a specific morphology for fibroblasts. The structure of the gel-like polymer layer was found to be not entirely appropriate for the efficient adhesion and formation of cell-to-cell junctions compared with dehydrated in full volume polymer nanolayers described in the literature. After proliferation, it was possible to separate cells from the polymer matrix simply by lowering the culture temperature. The polymer matrices were dissolved, and the cells lost their anchor surface. However, this pioneering approach to matrices for cell culture in vitro, with the application of their temperature-dependent solubility, requires further study, and development of a material that is not subjected to such significant hydration should be pursued.

## Figures and Tables

**Table 1:** Characteristics of the P(iPrOx-nPrOx) copolymer.

iPrOx:nPrOx	M_theoret_	M_n_ (g/mol)	*Ð*	DP	T_CP_ (°C)	T_g_ (°C)
47:53	52,000	51,000	1.32	450	27	50

**Table 2:** Thickness of P(iPrOx-nPrOx) films in the dry state and after incubation in water at 40 °C.

Sample Description	Film 1	Film 2	Film 3
In the dry state	50 µm ± 5 µm	185 µm ± 15 µm	20 µm ± 5 µm
After incubation in water at 40 °C	70 µm ± 15 µm	220 µm ± 20 µm	30 µm ± 5 µm
Increase in thickness/initial thickness	0.4	0.19	0.5

## Supplementary Materials

The following are available online at https://www.mdpi.com/1996-1944/13/12/2702/s1, Figure S1: Scheme presenting preparation of matrices., Figure S2: P(iPrOx-nPrOx) mold during shape deformation., Figure S3: AFM and SEM micrographs for Films 1 and 3 in dry state and after incubation in water at 40 °C for 2 h, Figure S4: SEM micrographs presenting a cross section of the P(iPrOx-nPrOx) films in a dry state and after incubation in water at 40 °C for 2 h The thickness of the polymer layer and silica slides (approximately 500 m) are marked in the images, Figure S5: FT-IR spectrum of P(iPrOx-nPrOx) specially dried in high vacuum, Figure S6: Morphology of fibroblasts after 72 h of cell culture with laminin on: Film 2 (**a**); and TCPS (**b**).
