# Supplementary material for: Poly(2-oxazoline) Matrices with Temperature-Dependent Solubility—Interactions with Water and Use for Cell Culture
Source: Materials (Basel). 2020 Jun 13;13(12):2702. doi: 10.3390/ma13122702 (PMC7344873; doi:10.3390/ma13122702)
Supplement: Supplementary file 1 [file materials-13-02702-s001.pdf]

materials

MDPI

Supplementary Materials

Poly(2-oxazoline) Matrices with Temperature-Dependent Solubility—Interactions with Water and Use for Cell Culture

Natalia Oleszko-Torbus, Marcelina Bochenek, Alicja Utrata-Wesołek, Agnieszka Kowalczuk, Andrzej Marcinkowski, Andrzej Dworak, Agnieszka Fus-Kujawa, Aleksander L. Sieroń and Wojciech Wałach

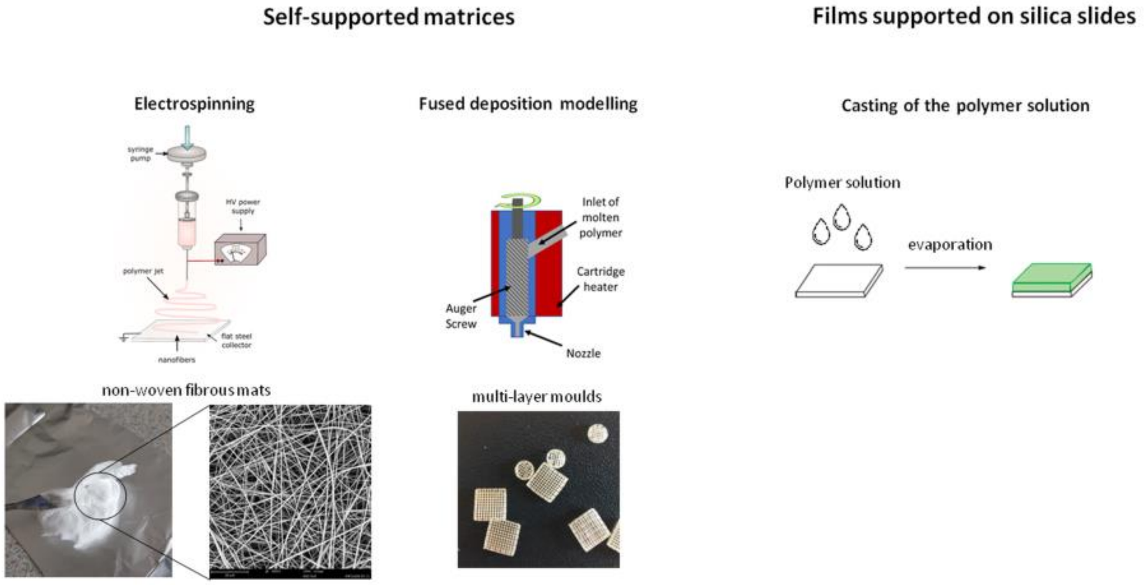

Figure S1. Scheme presenting preparation of matrices.

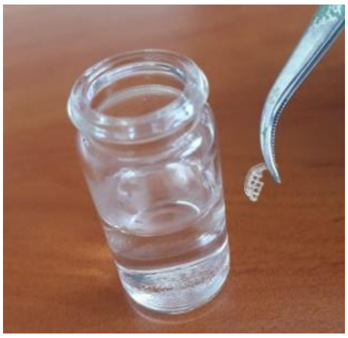

Dry P(iPrOx-nPrOx) mold

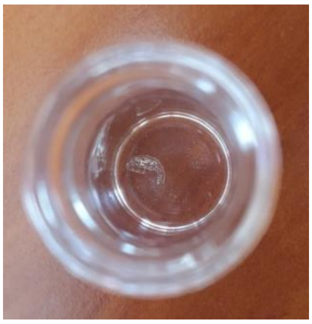

Mold incubated in water at 40 °C for several minutes

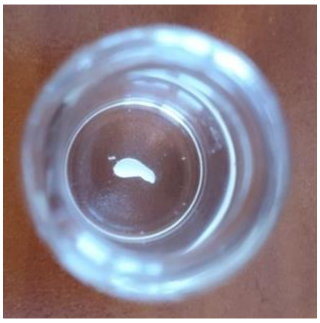

Mold after 2 h of incubation in water at 40 °C

Figure S2. P(iPrOx-nPrOx) mold during shape deformation.

Film 1 in the dry state

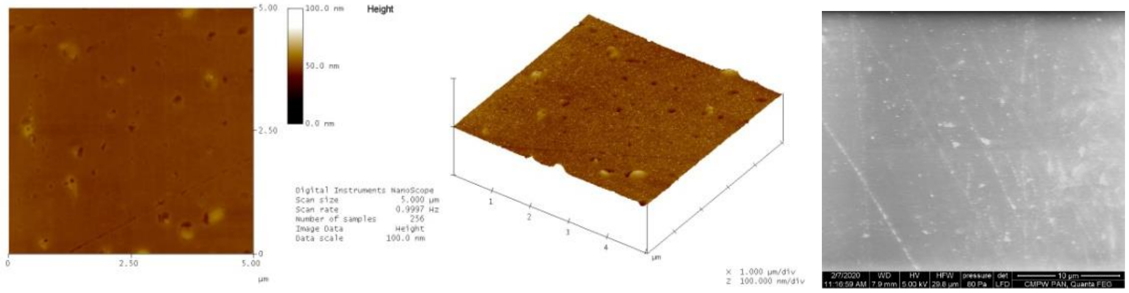

Film 1 after incubation

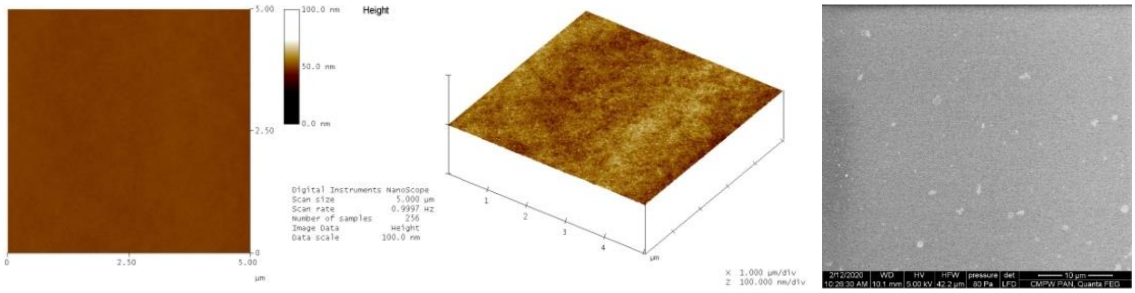

Film 3 in the dry state

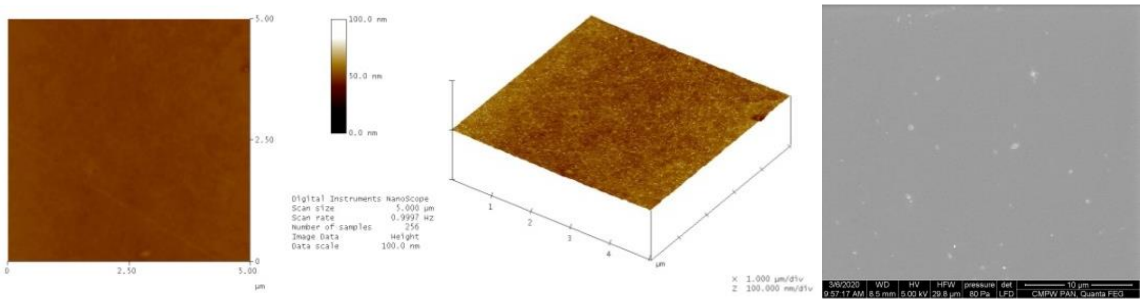

Film 3 after incubation

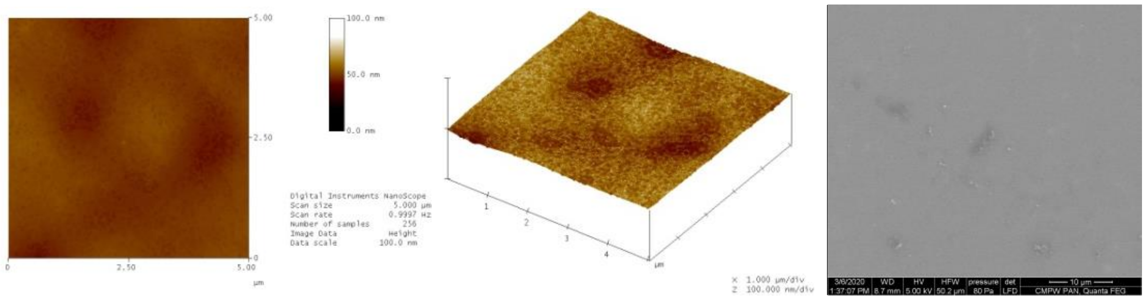

Figure S3. AFM and SEM micrographs for Films 1 and 3 in dry state and after incubation in water at 40 °C for 2 h.

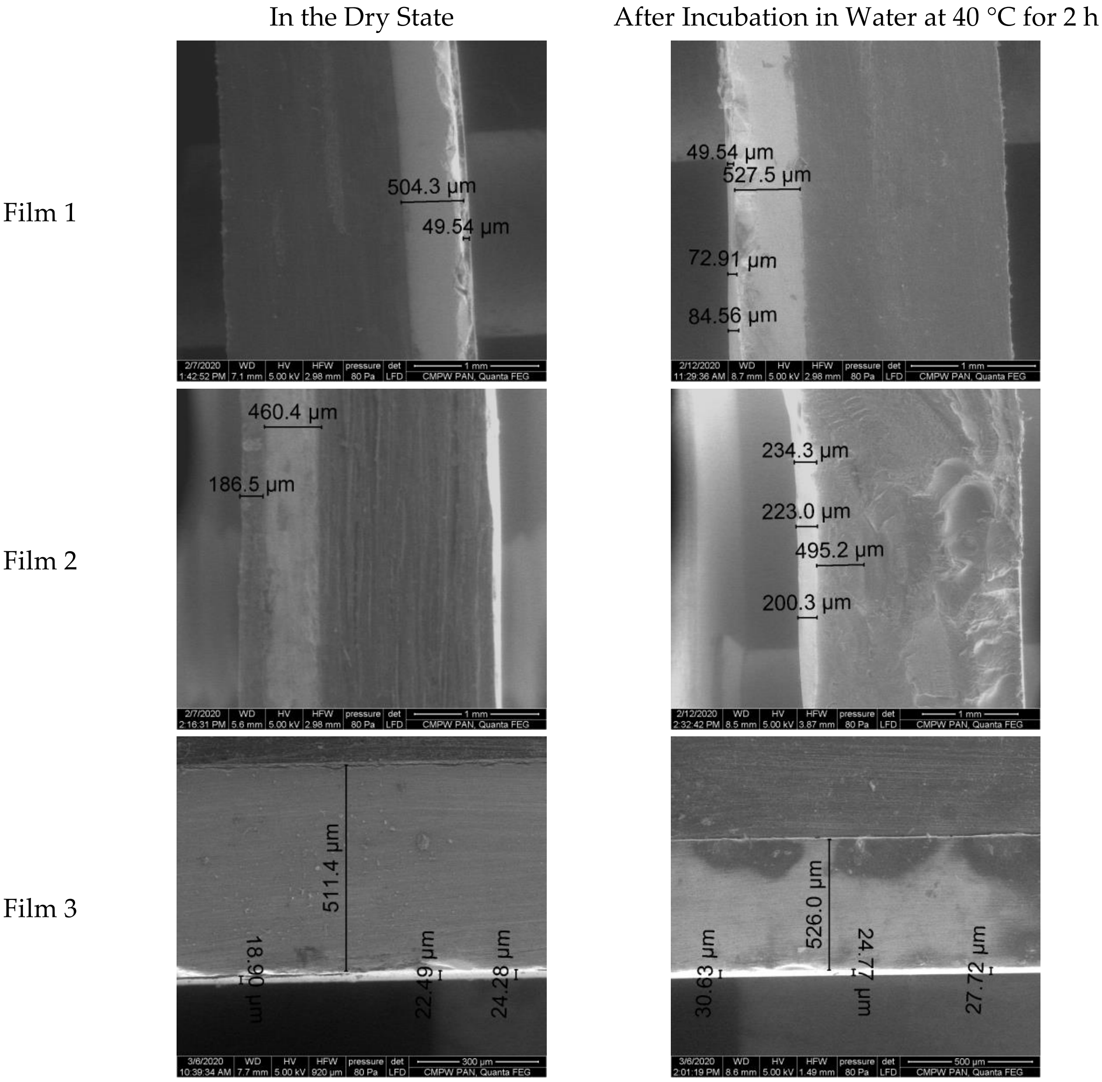

Figure S4. SEM micrographs presenting a cross section of the P(iPrOx-nPrOx) films in a dry state and after incubation in water at 40 °C for 2 h The thickness of the polymer layer and silica slides (approximately 500 m) are marked in the images.

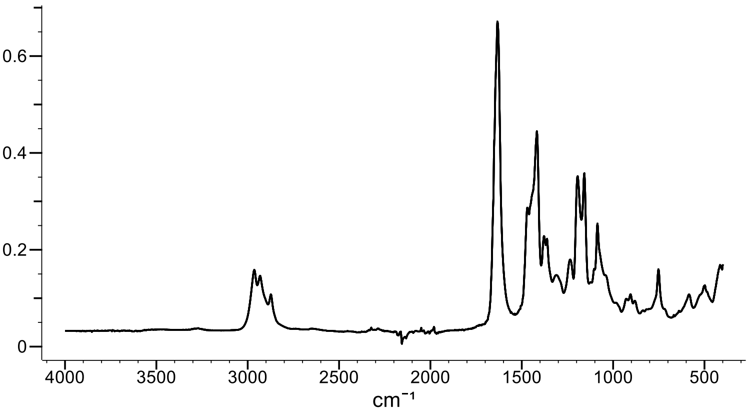

Figure S5. FT-IR spectrum of P(iPrOx-nPrOx) specially dried in high vacuum.

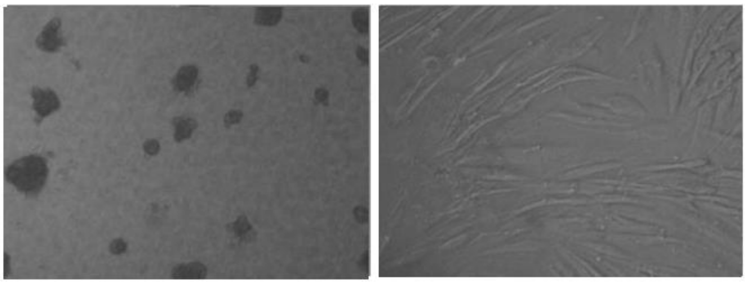

(**a**) (**b**)

Figure S6. Morphology of fibroblasts after 72 h of cell culture with laminin on: Film 2 (**a**); and TCPS (**b**).

© 2020 by the authors. Submitted for possible open access publication under the terms and conditions of the Creative Commons Attribution (CC BY) license (http://creativecommons.org/licenses/by/4.0/).
